# Calcium fluoride based multifunctional nanoparticles for multimodal imaging

**DOI:** 10.3762/bjnano.8.148

**Published:** 2017-07-18

**Authors:** Marion Straßer, Joachim H X Schrauth, Sofia Dembski, Daniel Haddad, Bernd Ahrens, Stefan Schweizer, Bastian Christ, Alevtina Cubukova, Marco Metzger, Heike Walles, Peter M Jakob, Gerhard Sextl

**Affiliations:** 1 Fraunhofer Institute for Silicate Research ISC, Neunerplatz 2, 97082 Wuerzburg, Germany; 2 Department of Chemical Technology of Materials Synthesis, University of Wuerzburg, Roentgenring 11, 97070 Wuerzburg, Germany; 3 MRB Research Center for Magnetic Resonance Bavaria, Am Hubland, 97074 Wuerzburg, Germany; 4 Department of Experimental Physics 5 (Biophysics), University of Wuerzburg, Am Hubland, 97074 Wuerzburg, Germany; 5 Translational Center Wuerzburg “Regenerative Therapies for Oncology and Musculosceletal Diseases”, Branch of Fraunhofer Institute for Interfacial Engineering and Biotechnology IGB, 97070 Wuerzburg, Germany; 6 University Hospital Wuerzburg, Chair Tissue Engineering and Regenerative Medicine, Roentgenring 11, 97070 Wuerzburg, Germany; 7 Magnetic Resonance and X-ray Imaging Department of Fraunhofer Development Center X-ray Technology EZRT, a division of Fraunhofer Institute for Integrated Circuits IIS, Am Hubland, 97074 Wuerzburg, Germany; 8 South Westphalia University of Applied Sciences, Luebecker Ring 2, 59494 Soest, Germany; 9 Fraunhofer Application Center for Inorganic Phosphors, Branch Lab of Fraunhofer Institute for Microstructure of Materials and Systems IMWS, Luebecker Ring 2, 59494 Soest, Germany

**Keywords:** calcium fluoride nanoparticles, magnetic resonance imaging (MRI), multifunctional nanoparticles, multimodal imaging, photoluminescence

## Abstract

New multifunctional nanoparticles (NPs) that can be used as contrast agents (CA) in different imaging techniques, such as photoluminescence (PL) microscopy and magnetic resonance imaging (MRI), open new possibilities for medical imaging, e.g., in the fields of diagnostics or tissue characterization in regenerative medicine. The focus of this study is on the synthesis and characterization of CaF_2_:(Tb^3+^,Gd^3+^) NPs. Fabricated in a wet-chemical procedure, the spherical NPs with a diameter of 5–10 nm show a crystalline structure. Simultaneous doping of the NPs with different lanthanide ions, leading to paramagnetism and fluorescence, makes them suitable for MR and PL imaging. Owing to the Gd^3+^ ions on the surface, the NPs reduce the MR *T*_1_ relaxation time constant as a function of their concentration. Thus, the NPs can be used as a MRI CA with a mean relaxivity of about *r* = 0.471 mL·mg^−1^·s^−1^. Repeated MRI examinations of four different batches prove the reproducibility of the NP synthesis and determine the long-term stability of the CAs. No cytotoxicity of NP concentrations between 0.5 and 1 mg·mL^−1^ was observed after exposure to human dermal fibroblasts over 24 h. Overall this study shows, that the CaF_2_:(Tb^3+^,Gd^3+^) NPs are suitable for medical imaging.

## Introduction

In recent years, medical imaging has become an important approach in the fields of diagnostics, therapy and regenerative medicine. Besides the classical technology of X-ray examination, contrast-rich methods such as computed tomography (CT), magnetic resonance imaging (MRI), positron emission tomography (PET) and ultrasonic techniques are being used increasingly for imaging soft tissue, e.g., cartilage imaging in progressive osteoarthritis. Advantages of different imaging techniques are used individually or combined to obtain a more detailed answer for medical questions and, thus, to reach a rapid and precise diagnosis. CT and MRI provide essentially morphological information and information on tissue structures and changes. Nuclear medicine procedures such as PET visualize metabolic processes and provide information on biochemical parameters. The optical imaging techniques such as fluorescence (PL) microscopy allow for a direct transfer of biological knowledge about cells in the in vivo application, e.g., endogenous regulation of transcription [1]. In this context, greater treatment success can be achieved through the combination of several detection methods. Contrast agents (CAs) are used to improve representation of structures and functions of the body by increasing the sensitivity and reducing the ambiguity in imaging techniques. Since the imaging techniques are based on different physical principles, different CAs are required. For the patient, this is associated with extended examination times, multiple injections and repeated contact with chemical substances. This results in an increased workload for the medical staff and an uncomfortable screening procedure for the patient. Therefore, it is desirable to develop a combined CA that is injected only once and then detected using different diagnostic methods with a higher comparability.

The production of CAs on the basis of nanoparticles (NPs) shows promise, as already examined on the infected myocardium [1–3]. Each imaging modality has its advantages and disadvantages. The integration of multiple functions into one NP system yields synergies and allows for a precise and fast diagnosis of diseases. Recently, various multimodal imaging probes on the basis of different functional NPs were fabricated for more accurate imaging and diagnosis [2]. One possibility is the synthesis of core/shell-structured NPs. Core and shell materials can be matched individually to specific detection methods. For example, the coating of a magnetic core with silicates or polymer shells doped with organic fluorophores or quantum dots (QDs) allows for the detection of NPs by MRI and PL [4]. Several successive shells can be designed of different inorganic materials. In this context, the following particle systems may be mentioned Gd_2_O(CO_3_)_2_·H_2_O/SiO_2_/Au, Fe_3_O_4_/C/Ag and Fe_3_O_4_/SiO_2_/Y_2_O_3_:(Yb^3+^,Er^3+^) core/shell NPs [5–7]. Another possibility is to create multifunctional NPs by precipitation and simultaneous doping of the NP matrix with various ions [8–9]. Due to their co-doping with lanthanide ions, NPs on the basis of calcium phosphate or gadolinium oxide are also detectable by MRI and PL [10–13].

In recent years, fluorides have attracted considerable interest owing to their unique optical properties [14]. Fluoride NPs were used in lighting, optical amplification and lasing [14] and are well-known strategic materials in optical and photonic technologies in general. Furthermore, they combine high quantum efficiency with favorable chemical and mechanical properties. They seem to be perfect materials as fluorescence host matrix owing to their low phonon energies and they subsequently minimize the quenching of the excited state of rare-earth ions. In contrast to chloride or bromide hosts, fluorides are completely air stable materials [15–16].

Other than fluoride NP systems doped with rare earth elements, such as LaF_3_:Ln^3+^, CeF_3_:Tb^3+^, NaYF_4_:(Yb^3+^,Er^3+^), NaGdF_4_:(Yb^3+^,Er^3+^), which were actively investigated during last decades for biomedical applications [17–22], alkaline earth metal fluorides such as CaF_2_ received little attention. There are only sporadic suggestions for the synthesis and application of this NP system as a labeling material. To date, CaF_2_ has attracted most attention with respect to UV lithography, UV-transparent optical lenses, the surface conditioning of glass, the promotion of biocompatible agents for bone and teeth reconstruction [23]. Calcium fluoride exhibits a wide transparent spectral window (190–1100 nm), large band gap (approx. 12 eV), low refractive index and low phonon energy [14].

Because of the high stability and flexibility of the fluorite structure, a number of various ionic substitutions can also be integrated in the CaF_2_ lattice [24]. Various methods have been reported for the preparation of rare-earth doped CaF_2_ NPs such as co-precipitation [14–15,24–26], hydrothermal methods [27–29], flame synthesis [30], microemulsion methods [31–32] and a fluorolytic sol–gel process [33]. The stability and biocompatibility of CaF_2_ makes it an attractive material for biomedical applications [28–29]. In addition, due to the high capacity to accept lanthanide ions, CaF_2_ is suitable for the preparation of CAs for multimodal imaging [24].

In this study, we report on synthesis and characterization of multifunctional NPs based on CaF_2_. These NPs are produced by wet-chemical synthesis and doped with multiple ions leading to paramagnetism and fluorescence, making them suitable for *T*_1_-weighted MRI and PL microscopy. The characterization of the resulting NPs is carried out by using transmission electron microscopy (TEM), X-ray diffraction (XRD) analysis, inductively coupled plasma optical emission spectrometry (ICP-OES), and photoluminescence (PL) spectroscopy. The capability of these NPs to be used as positive CAs for MRI was also investigated. In addition, the cytotoxicity of the NPs was tested by a cell culture based viability assay.

## Results and Discussion

### Synthesis and characterization of the multifunctional nanoparticles

The synthesis of the CaF_2_:(Tb^3+^,Gd^3+^) NPs was carried out in analogy to the reported wet-chemical procedure that is based on a co-precipitation process in ethanol [26]. Moreover, the NPs were doped with different lanthanide ions (Tb^3+^, Gd^3+^; 1 mol % based on Ca content) to guarantee a PL and MR activity. CaCl_2_, Tb(NO_3_)_3_·5H_2_O, GdCl_3_·6H_2_O and NH_4_F were used as reactants to prepare the NPs by a low-temperature single-step approach. CaCl_2_ and NH_4_F exhibit a significant solubility in water, but CaF_2_ is insoluble in water and precipitates from aqueous solution. It is difficult to control the particle growth in aqueous solution and therefore the synthesis of the doped NPs is carried out in ethanol. This solvent contains a very low F^−^ ion concentration because of the low solubility of NH_4_F in an ethanol solution and therefore the particle growth is slower [26].

**Figure 1:**
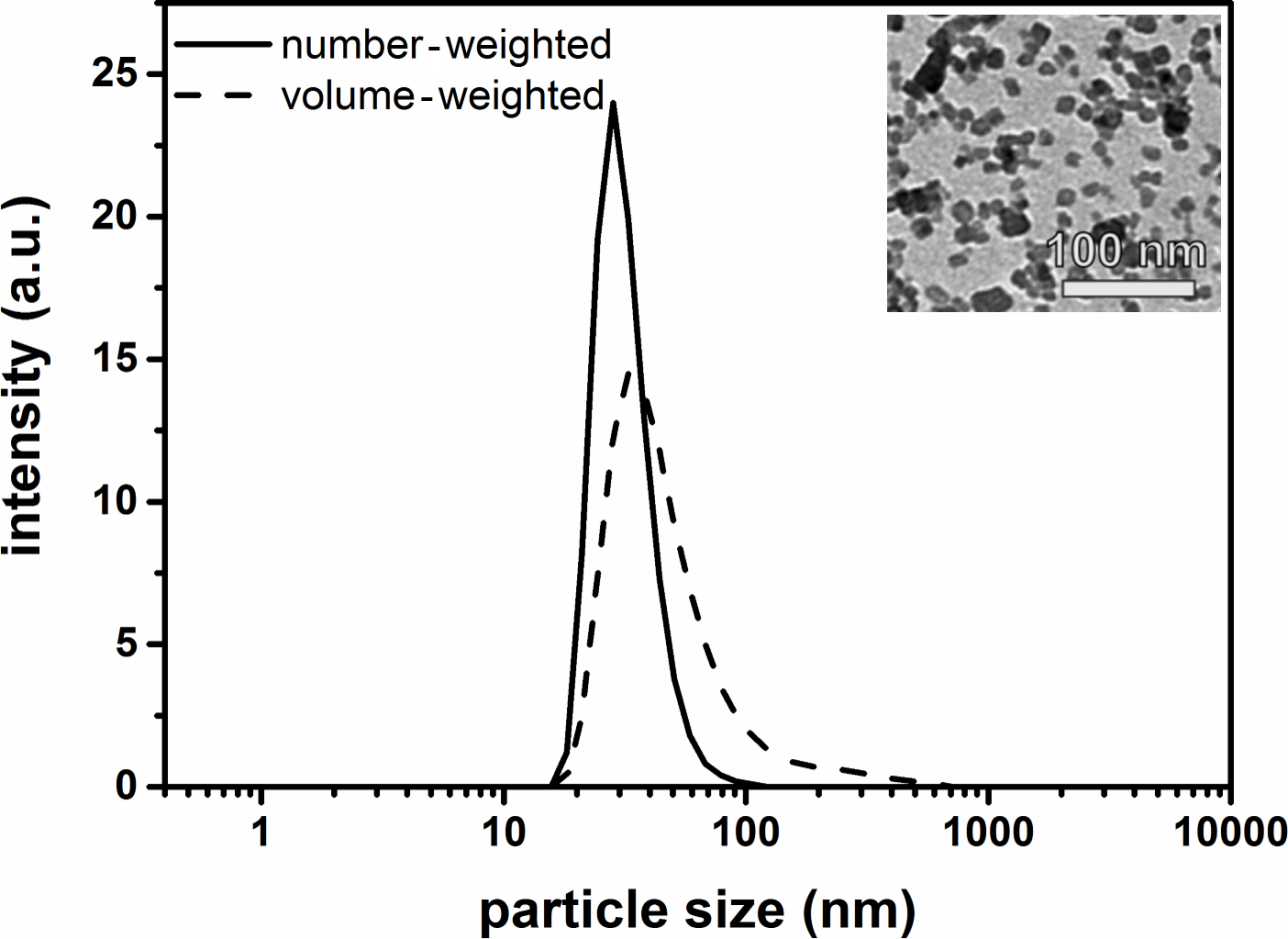
DLS measurement of the CaF_2_:(Tb^3+^,Gd^3+^) NPs (number- and volume-weighted). Inset: TEM micrograph of the same particles. The size of the NPs is in the range of 5–10 nm and they have a spherical shape.

The inset in Figure 1 shows the TEM micrograph of the CaF_2_:(Tb^3+^,Gd^3+^) NPs. The NPs possess a spherical shape with an average diameter of 5–10 nm. The results of the dynamic light scattering (DLS) show that these NPs are non-agglomerated and exhibit a narrow size distribution and a hydrodynamic particle diameter of 25–30 nm (number- and volume-weighted, Figure 1, see also DLS of the stabilized NPs, Figure S1, Supporting Information File 1 (number-weighted)).

For the determination of the MR relaxivity of the NPs, we use the hydrodynamic particle diameter from the DLS, because the correlation time between them and the surrounding water molecules depends on the tumbling of the NPs, which is influenced by their size and their morphology.

**Figure 2:**
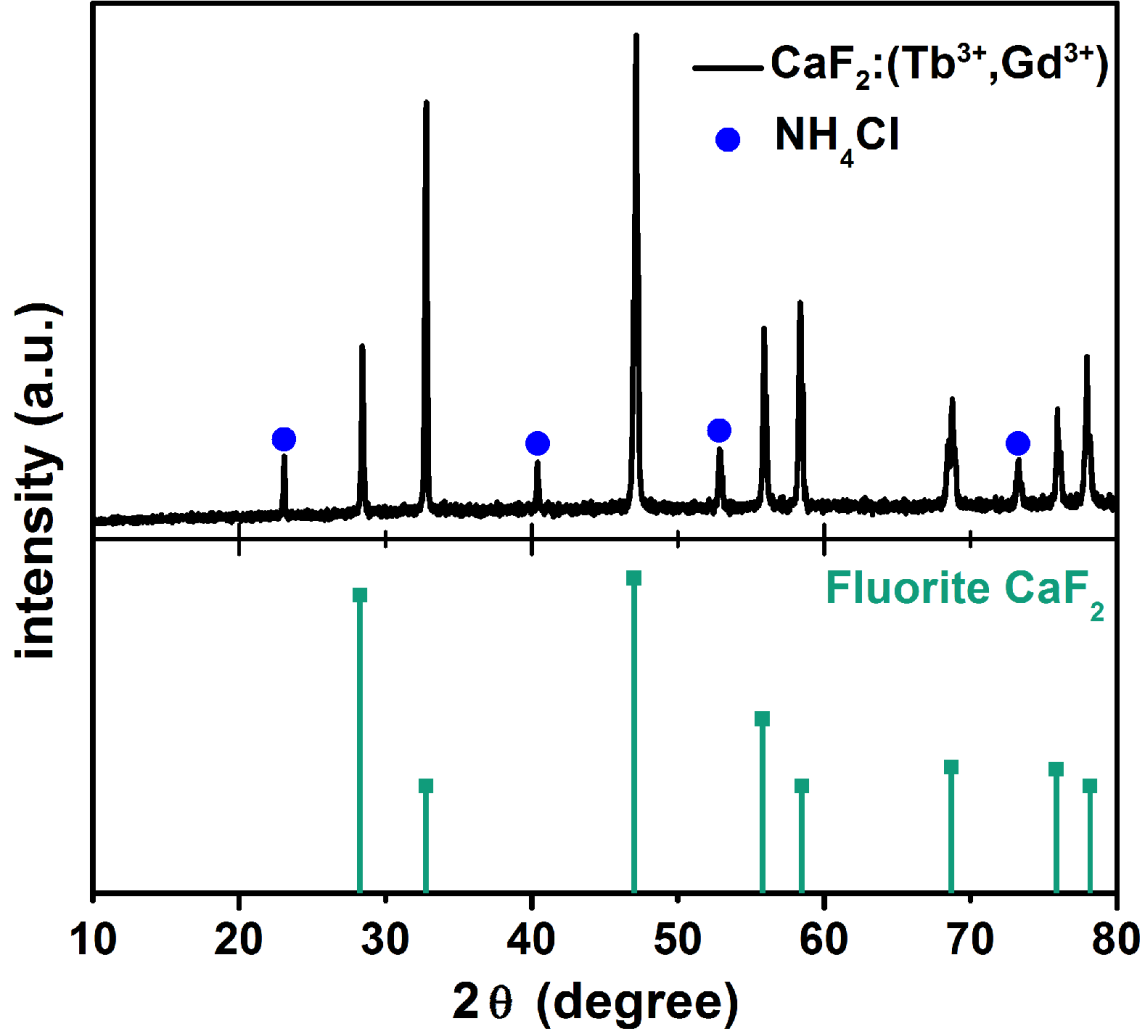
In the upper part, the XRD pattern of the CaF_2_:(Tb^3+^,Gd^3+^) NPs (*d* = 5–10 nm, doping concentration of Tb^3+^ and Gd^3+^: 1 mol %) is plotted. Below a reference spectrum from the database JCPDS (Joint Committee on Powder Diffraction Standards, Powder Diffraction File: 035-0816) is shown. The reflexes of both spectra appear at the same diffraction angles 2θ, which indicates the crystalline structure (CaF_2_ = fluorite) of the NPs. The blue points mark the peaks of NH_4_Cl.

Figure 2 displays a selected XRD pattern of the crystalline CaF_2_:(Tb^3+^,Gd^3+^) NPs, all other samples exhibit the same tendency. The phase analysis indicates that the obtained product shows prominent peaks well accordant with the JCPDS standard card (Joint Committee on Powder Diffraction Standards, Powder Diffraction File: 035-0816) of fluorite (CaF_2_). Moreover, there are reflexes of NH_4_Cl detectable which come from the educts. Doping with multiple ions does not have influence on the formation of calcium fluoride crystal lattice.

Since the XRD measurement only implies that the NPs have the crystalline structure of fluorite and nothing about the incorporation of the lanthanide ions, we analyze the composition of our nanoparticles further by means of ICP-OES. Table 1 shows the outcome of a representative sample.

**Table 1:** Representative outcome of an ICP-OES measurement of CaF_2_:(Tb^3+^,Gd^3+^) NPs.

element	amount of substance (mol)	doping (mol %)

F	0.46	
Ca	0.20	
Tb	0.02	0.87
Gd	0.02	0.92

The obtained ratio of calcium and the lanthanide ions to fluoride ((Ca+Ln)/F = 0.52) is an additional confirmation of the crystalline structure of fluorite with a composition of CaF_2_. The doping levels are in the intended range of 1 mol %. However, there is a lower content of Tb^3+^ and Gd^3+^ (total amount of 1.83 mol %), which indicates a higher reaction rate of calcium compared to the dopants. This could be correlated to the different ion radii.

### Photoluminescence spectroscopy

**Figure 3:**
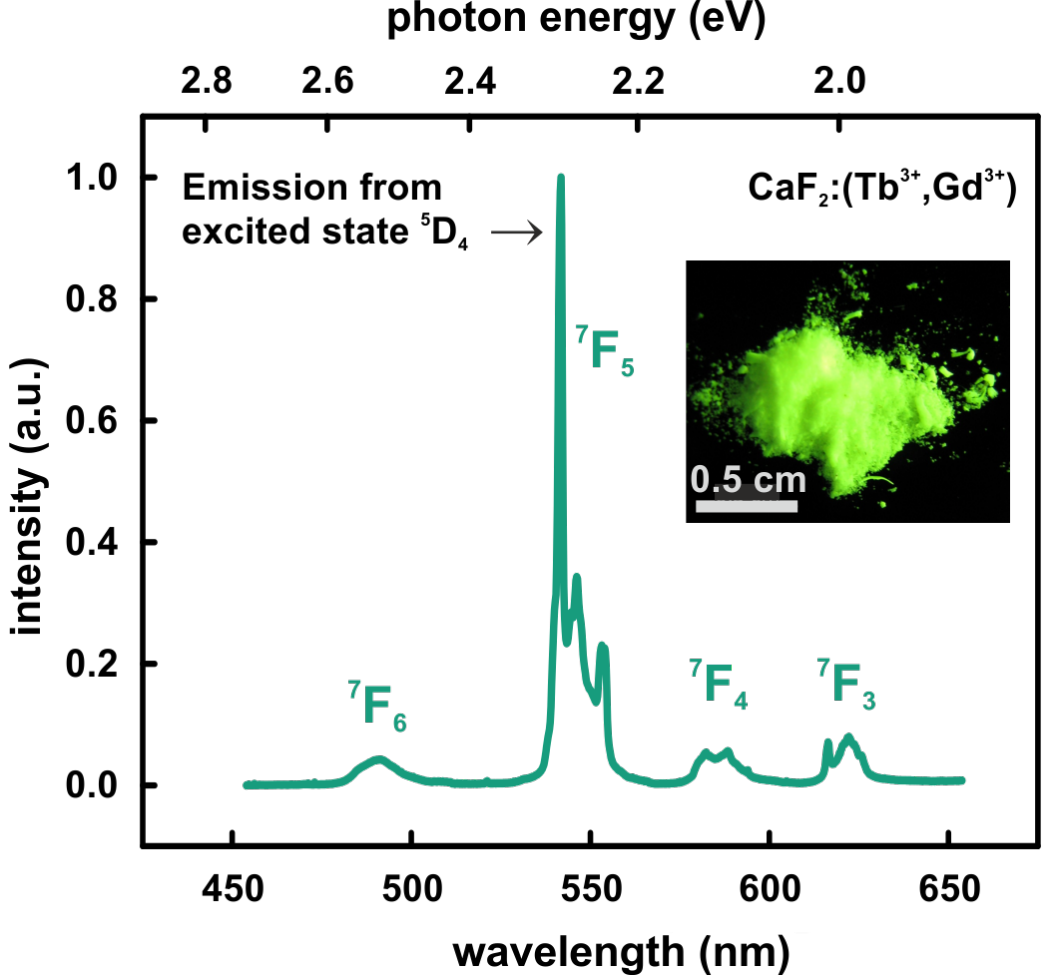
Normalized photoluminescence spectrum of CaF_2_:(Tb^3+^,Gd^3+^) NPs at an excitation wavelength of λ_exc_ = 254 nm. The emission spectrum shows several maxima (490, 542, 586 and 622 nm), which represent the Tb^3+^-related transitions from the ^5^D_4_ excited state to the energy levels indicated. The maximum intensity occurs in the green spectral range at a wavelength of λ = 542 nm. Inset: CaF_2_:(Tb^3+^,Gd^3+^) NP powder under UV light excitation (λ_exc_ = 254 nm). The green luminescence matches the maximum of the emission spectrum.

With respect to a later usage of our NPs as a contrast agent for PL we have also investigated the optical properties. Since terbium and its optical properties are extensively described in the literature [34–37], we use it as a model system in order to proof the integration of the ions in the calcium fluoride lattice (proof of principle). For a later clinical usage certainly we have to exchange terbium for a NIR dye or something similar because of the high sensitivity of living tissues towards UV light. In Figure 3 the emission spectrum of CaF_2_:(Tb^3+^,Gd^3+^) NPs at an excitation wavelength of λ_exc_ = 254 nm is shown. There are several maxima (490, 542, 586 and 622 nm), which represent the Tb^3+^-related transitions from the ^5^D_4_ excited state to the energy levels indicated [34–35]. The main emission line can be assigned to the ^5^D_4_→^7^F_5_ transition of Tb^3+^ and causes an intense emission in the green spectral range (λ = 542 nm, Figure 3) [36–37]. Additionally, to XRD and ICP-OES measurements this was a confirmation of a successful integration of the Tb^3+^ ions in the calcium fluoride host lattice.

### Magnetic resonance imaging

MRI is a non-invasive method that is optimized for soft tissue imaging in daily clinical use. Paramagnetic CAs are often used to reduce the measurement period or to gain higher signal-to-noise-ratios (SNR) which allows for improved diagnosis. Within this study, the capability of CaF_2_:(Tb^3+^,Gd^3+^) NPs to be used as positive CAs for MRI was investigated. To this end, different NP samples dispersed in water were characterized by MRI. To determine the contrast effect, NP dispersions of various concentrations (0.4–18.2 mg·mL^−1^) were analyzed.

#### CaF_2_:(Tb^3+^,Gd^3+^) NPs as MRI contrast agent

Doping with Gd^3+^ ions leads to paramagnetism of the CaF_2_ NPs. Because of this the NPs can be used as a *T*_1_ CA. Additionally, there should be also an attenuation of the CT signal, which allows for the application as a CT CA. This property is already closer investigated in an ongoing study and will be shown in an additional publication in the future.

**Figure 4:**
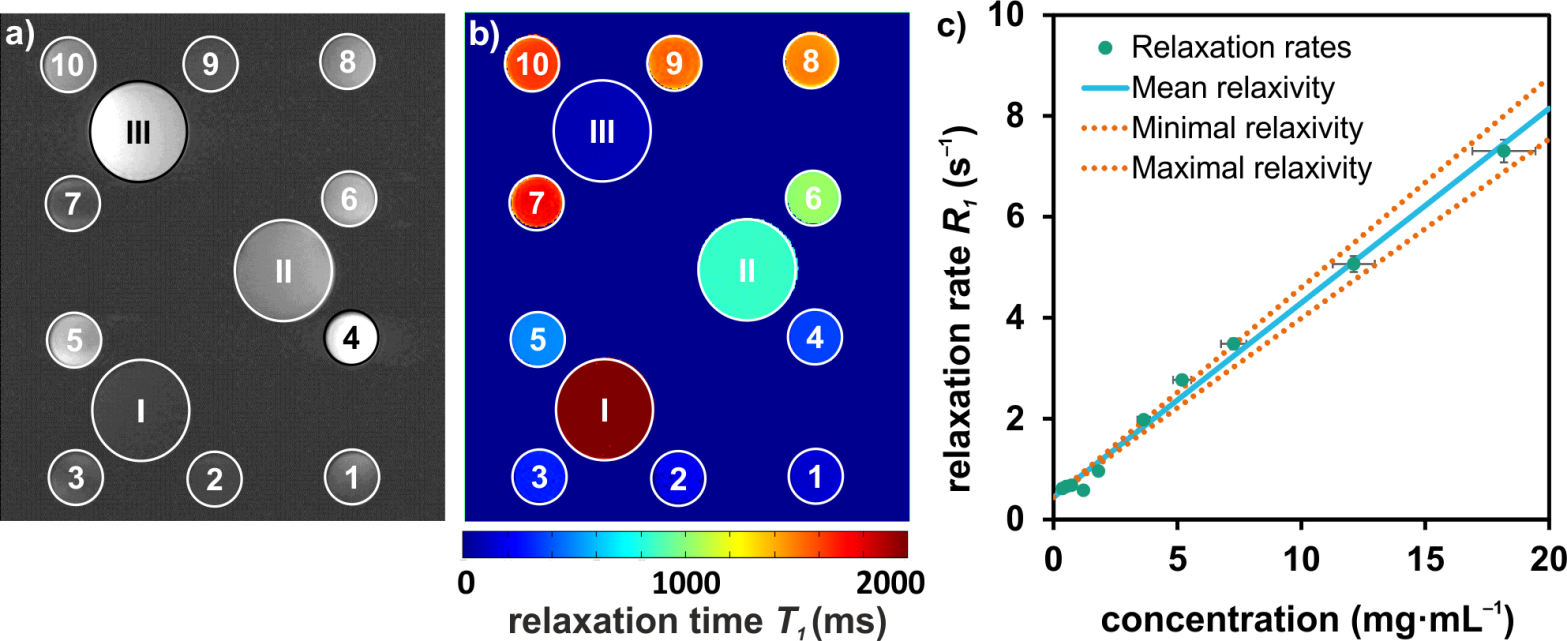
a) *T*_1_-weighted MR image of the CaF_2_:(Tb^3+^,Gd^3+^) NPs with different concentrations in the range from 0.4 to 18.2 mg·mL^−1^ (1–10). I–III are reference samples of water and two concentrations of Magnevist. The different concentrations result in observable differences of the signal intensities. In b) the following *T*_1_-map with a corresponding color range from 0 to 2000 ms is shown. c) The relaxivity (*r* = 0.385 ± 0.030 mL·mg^−1^·s^−1^) arises from the slope by plotting the relaxation rates over the concentrations. The errors of the relaxivity are given by the minimal and maximal slope.

The *T*_1_-weighted image of the CaF_2_:(Tb^3+^,Gd^3+^) NPs with concentrations in the range from 0.4 to 18.2 mg·mL^−1^ is shown in Figure 4a. Due to the different concentrations of the samples, the *T*_1_ relaxation time constants vary and therefore, different signal intensities are observable at different time points.

To evaluate the potential CA not only on a qualitative basis, it is required to determine the efficiency quantitatively. First, it is necessary to measure the signal intensity at different time points and fit these intensities with a mono-exponential function. A *T*_1_-map can be calculated (cf. Figure 4b). In this batch, the *T*_1_ values of the NPs vary from 137 to 1633 ms with decreasing concentrations. Plotting the relaxation rate *R*_1_ (inverse relaxation time *T*_1_) over the concentration of the samples, the relaxivity *r* arises from the slope of the linear fit (cf. Equation 1, Figure 4c).

[1]
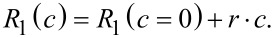


The relaxivity indicates the efficiency of the CA. The most common CA in clinical applications is Magnevist (gadopentetate dimeglumine) with a relaxivity of 4.89 mL·mg^−1^·s^−1^ [38]. In this study, Magnevist was used as a reference in each measurement. Generally, the relaxivity is given in liters per mole per second. To compare the obtained relaxation rates from our NP dispersions with the relaxation rate from Magnevist we should convert the units because Magnevist is a complex with only one Gd^3+^ ion. In contrast, there are many Gd^3+^ ions in one NP evoking the MR activity. Unfortunately, we cannot quantify by now the exact amount of Gd^3+^ ions on the surface. The ICP-OES measurements (cf. Table 1) tell us how many Gd^3+^ ions are within the NPs in total, but most probably only the Gd^3+^ on the surface are responsible for the contrasting effect. Therefore, we convert the units into milliliters per milligram per second (for the calculation see Figure S2, Supporting Information File 1).

The relaxation rates obtained have a standard deviation of 3.1%. This value is used for the uncertainty of the MRI measurement itself and therefore also for the relaxation rates of the NPs. For acquiring the relaxivity of the NPs, an additional source of error is the uncertainty of the concentration of each sample. Determining the concentration, a gravimetrical measurement was carried out three times for each sample. The maximum deviation of the values was about ±7%, because of different error sources such as weighing or pipetting of the small sample volumes. The error of the relaxivity is given by the resulting minimal and maximal slope (Figure 4c).

### Reproducibility of the MR relaxivity

**Figure 5:**
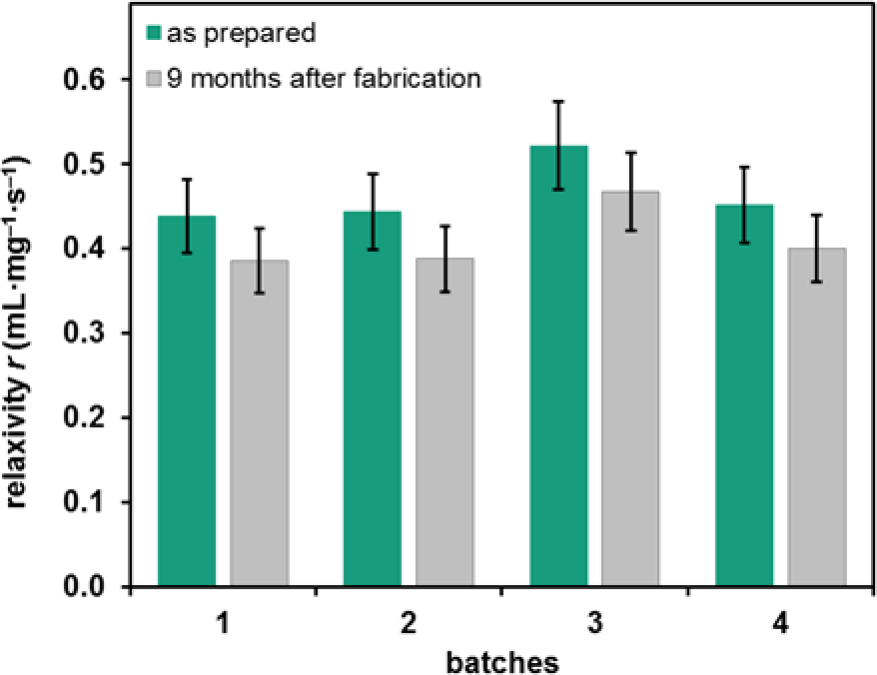
Relaxivity values of the four batches as prepared (green) and nine months (grey) after fabrication. Considering the error bars, the measured relaxivities directly after the preparation overlap with each other. After nine months a decrease of the relaxivity of each batch can be observed.

To test the reproducibility of the CA efficiency, four batches of NPs were produced and their relaxivities were determined. These results are shown in Table 2 and Figure 5.

**Table 2:** Relaxivities of four different batches: as prepared (row 1) and nine months after fabrication (row 2).

	batches	1	2	3	4

relaxivity *r* [mL·mg^−1^·s^−1^]	as prepared	0.438 ± 0.044	0.443 ± 0.044	0.522 ± 0.052	0.451 ± 0.045
	nine months after fabrication	0.385 ± 0.038	0.388 ± 0.039	0.467 ± 0.046	0.400 ± 0.040

The first row of Table 2 and the green bars of Figure 5 represent the results of the measurements directly after fabrication. All results overlap with their error bars and additionally the mean value lies also within the ranges of all batches. Therefore, the relaxivities of all batches are comparable with each other indicating a high reproducibility of the synthesis procedure. This matches to the above described results of TEM, DLS and ICP-OES examinations.

### Long-term stability

The long-term stability of the relaxivity over time was examined. All batches were investigated nine months after fabrication. These results are also shown in Table 2 and Figure 5. It is apparent that all relaxivities decrease over time. The difference between both groups (all batches as prepared and nine months after fabrication), which was tested with a *t*-test, is highly significant (*p* < 0.01). This decrease of the relaxivities cannot be explained with the deviation of the MR measurements. More likely, this trend is triggered by an agglomeration of the NPs. This results in a lower concentration of Gd^3+^ on the surface of the NPs, which leads to a lower interaction with the surrounding protons, implying a higher relaxation time constant and consequently a lower relaxivity. On average, all batches decrease about 11.6% after nine months. Through an examination of the vertical distribution of the *T*_1_ relaxation time constants in the probing tubes a sedimentation of the NPs or a decrease of the relaxivity can be excluded. Also, a decomposition of the NPs, resulting in an increase of the free Gd^3+^ concentration within the solution and therefore an increase of the relaxivity, does not take place. This is another very important property of our CA, because of the toxicity of free Gd^3+^ ions.

### Biocompatibility

**Figure 6:**
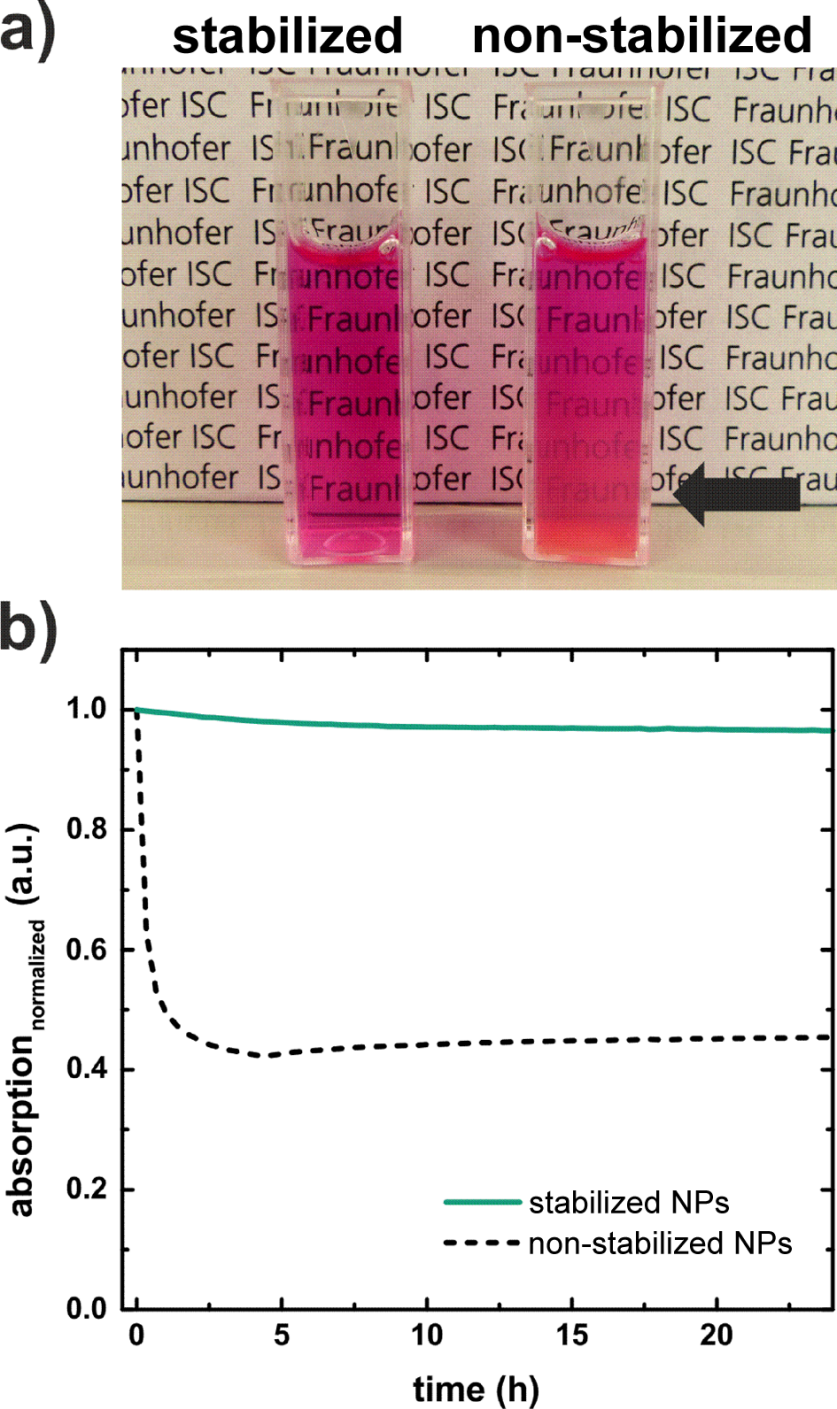
Sedimentation study of the CaF_2_:(Tb^3+^,Gd^3+^) NPs (5 mg·mL^−1^) in Dulbecco’s Modified Eagle’s Medium (DMEM) with 10% FCS stabilized with Melpers^®^2450 and non-stabilized: a) photographs of the stabilized NPs and the non-stabilized NPs 24 h after dispersing the NPs in FCS-containing cell-culture medium and b) absorbance measurement (λ_abs_ = 700 nm) of the samples over a period of 24 h.

In general, NPs without appropriate surface modification have a disposition to agglomerate and sediment subsequently under physiological conditions because of their pH value and salt content [39–41]. One crucial requirement for the application of NPs in cell-culture experiments or animal testing is the stabilization in physiological media. In contrast to an electrostatically stabilization of the NPs, for example by capping the CaF_2_ NPs surface with citrate groups [28], we ensure the stability of the NPs in serum-containing cell-culture media in an electrosterical way. To this end, a polymer consisting of a polycarboxylate ether backbone and polyethylene oxide side chains bound to the backbone as esters (Melpers^®^2450), which can be considered as non-toxic [42], is adsorbed via Coulomb attraction between the negatively charged backbone on the positively charged NP surface [43]. As shown in photographs of bare and Melpers^®^2450-stabilized NPs dispersed in cell-culture medium containing fetal calf serum (FCS) (cf. Figure 6a), bare NPs start to sediment after 24 h and the stabilized sample remains clear. Additionally, the colloidal stability was monitored by UV–vis spectroscopy. The absorbance measurements (λ_abs_ = 700 nm) of stabilized and non-stabilized CaF_2_:(Tb^3+^,Gd^3+^) NPs in FCS-containing cell-culture medium over a period of 24 h is shown in Figure 6b. In contrast to non-stabilized NPs, the stabilized sample shows hardly any change in absorbance over a period of 24 h. The measured curve of non-stabilized NPs decreases within 2 h because of light scattering on NP agglomerates. Light-microscopy images of dispersions of stabilized and non-stabilized CaF_2_:(Tb^3+^,Gd^3+^) NPs in FCS-containing cell-culture medium are given in Figure S3 (Supporting Information File 1).

**Figure 7:**
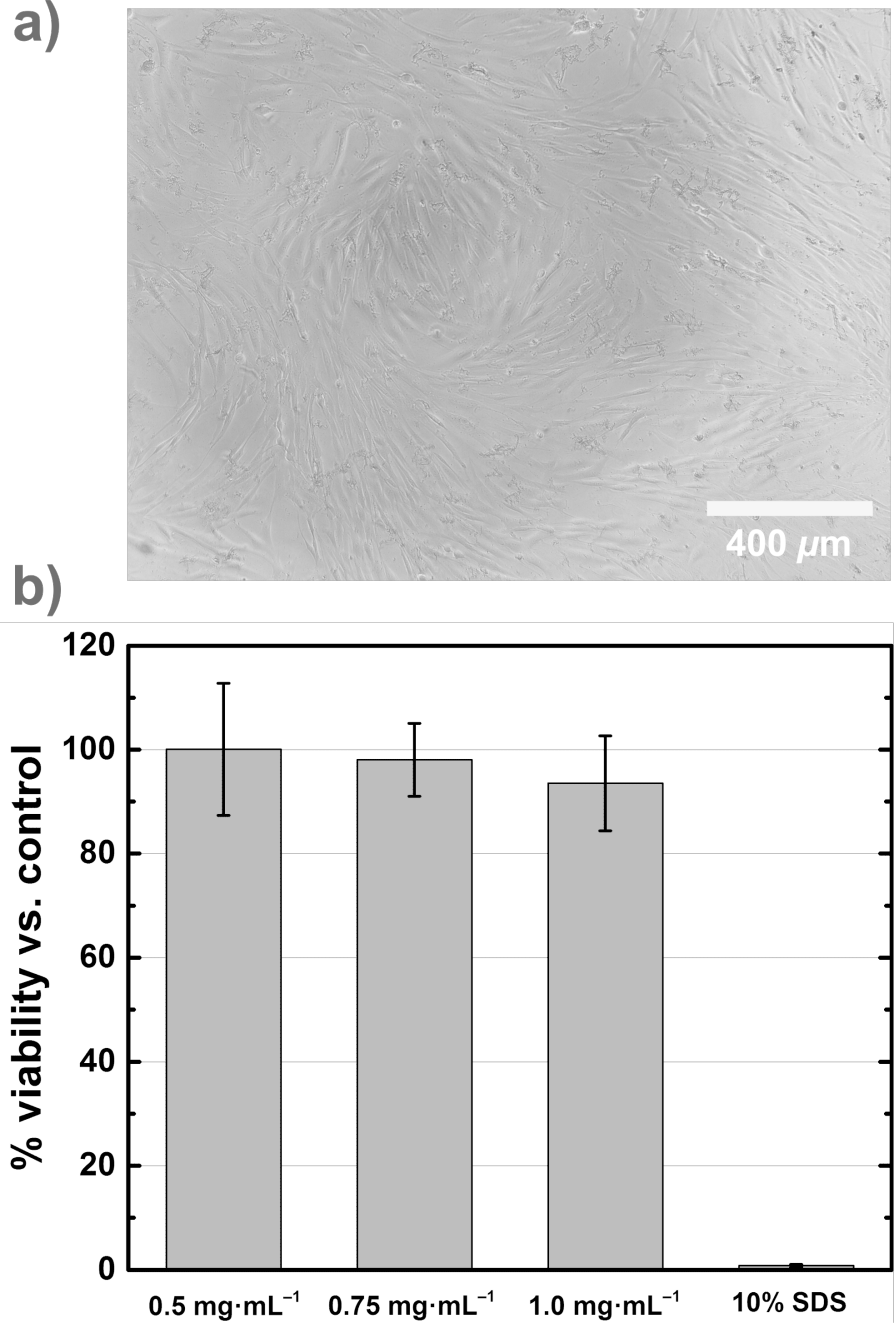
a) Representative microscopic image of hdF 24 h after treatment with the NPs (*c* = 1 mg·mL^−1^). b) Cell viability 24 h after adding CaF_2_:(Tb^3+^,Gd^3+^) NPs at concentrations between 0.5 and 1 mg·mL^−1^ to hdF. 10% SDS was used for the positive control. All samples have cell viabilities over 80% and therefore the NPs can be classified as non-cytotoxic (*n* = 3).

Finally, the viability of human dermal fibroblasts (hdF) after treatment with the NPs stabilized with Melpers^®^2450 for 24 h was evaluated. Therefore, the CellTiter-Glo assay was used [44], a method that is based on the quantification of adenosine-5′-triphosphate (ATP), which signals the presence of metabolically active cells. Adding the CellTiter-Glo reagent directly to hdF results in cell lysis and generates a luminescent signal directly proportional to the amount of the ATP concentration. The particle samples with a cellular viability over 80% can be classified as biocompatible. We have chosen concentrations of CaF_2_:(Tb^3+^,Gd^3+^) NPs between 0.5 and 1 mg·mL^−1^ for the assay because in this concentration range we have observed a good MR activity. Figure 7a shows a representative microscopic image of the hdF 24 h after treatment with the NPs (*c* = 1 mg·mL^−1^). There is clear evidence that the cells treated with the NPs compared to untreated cells kept their typically morphology and proliferated normally under standard culture conditions. The granular structures in the picture arise from FCS (cf. untreated cells, Figure S4, Supporting Information File 1). The results of a CellTiter-Glo assay show the viability of hdF 24 h after treatment of these cells with the NP dispersions (cf. Figure 7b). NP concentrations of 0.5, 0.75 and 1.0 mg·mL^−1^ yield cell viabilities of more than 80% with respect to the positive control. Thus in this concentration range no cytotoxicity of CaF_2_:(Tb^3+^,Gd^3+^) NPs is observed on hdF.

## Conclusion

In summary, we have demonstrated a new multifunctional particle system CaF_2_:(Tb^3+^,Gd^3+^), which was fabricated via a co-precipitation process. TEM, DLS, XRD and ICP-OES examinations deliver a consistent characterization of the NPs. According to TEM and DLS measurements, the mean size of the NPs is in the range of 5–10 nm and they have a spherical shape. In the XRD diffractogram the crystalline structure of fluorite CaF_2_ is observable. The outcome of ICP-OES shows a congruent composition of the NPs in all different batches. Hence the results of these characterization methods evidence that the used synthesis was successful and capable of producing the desired particle system. The assumption is that doping with the rare-earth ions Tb^3+^ and Gd^3+^ leads to a PL and MR activity. The Tb^3+^ emission spectrum shows maxima at the expected wavelengths (489, 542, 585, 621 and 667 nm). This signifies the successful Tb^3+^ doping and thus, the NPs are suitable for use as a PL CA. With the second investigated imaging technique MRI we verified the integration of the Gd^3+^ ions in the CaF_2_ lattice and the reproducibility of the NP synthesis procedure. Furthermore, we investigated the long-term stability of the relaxivities. In fact, the results for all batches show a decrease of the relaxivity of about 11.6% after nine months. Finally, the cell viability of the NPs stabilized with Melpers^®^2450 was evaluated in hdF and we can show that the NP system is biocompatible and non-toxic.

Overall, we have developed a very promising particle system CaF_2_:(Tb^3+^,Gd^3+^), which can be used as a multimodal CA for two different imaging methods and therefore allows for a more reliable, precise and time efficient diagnosis of diseases.

## Experimental

### Materials

Calcium chloride (CaCl_2_, ≥95%), ammonium fluoride (NH_4_F, p.a.), terbium(III) nitrate pentahydrate (Tb(NO_3_)_3_·5H_2_O, 99.9%) and gadolinium(III) chloride hexahydrate (GdCl_3_·6H_2_O, 99%) were purchased from Sigma-Aldrich and used without further purification.

### Synthesis of CaF_2_:(Tb^3+^,Gd^3+^) NPs

Following Wang et al., 3.72 g (33.5 mmol) CaCl_2_, 146 mg (340 μmol, 1 mol % based on Ca content) Tb(NO_3_)_3_·5H_2_O and 121 mg (340 μmol, 1 mol % based on Ca content) GdCl_3_·6H_2_O were dissolved in 420 mL ethanol [26]. 2.5 g (67.5 mmol) of ammonium fluoride were added under sonification. Subsequently, the solution was stirred at room temperature for 12 h. The resulting precipitate was collected by centrifugation and washed with ethanol and deionized water for three times to remove possible impurities such as CaCl_2_. Then the precipitate was dried at 60 °C for 12 h and collected for characterization.

### Characterization

The morphology of the NPs was studied by TEM on a Zeiss EM 900 transmission electron microscope at an acceleration voltage of 200 kV. Samples were prepared by dipping 200 mesh copper grids coated with a thin carbon film (Quantifoil Micro Tools GmbH) into aggregate-free NP dispersions. The size of the particles was determined by the measurement tools of Fiji. The DLS was measured with a Zetasizer Nano-ZS from Malvern Instruments. The DLS measurement was carried out in aqueous solutions. X-ray diffraction measurements were carried out on a Phillips PW 1730/10 employing Cu Kα radiation. The composition of the NPs was determined by ICP-OES using a Varian Wista Pro spectrometer. The crystallinity of the powder samples was analyzed with a Philips PW 1152. For photoluminescence measurements, a custom-built photospectrometer (S&I Spectroscopy & Imaging FluoroVista) was used. The excitation of the Tb^3+^-related emission spectra was carried out with a 254 nm UV lamp (Vilber Lourmat VL-4.LC), the emission was detected with a high-speed silicon CCD camera (Princeton Instruments PIXIS256). The spectra were not corrected for the spectral sensitivity of the experimental setup.

### MRI measurements

To guarantee a homogenous distribution within each sample, all tubes were sonicated for five minutes and vortexed afterwards. The MRI examinations took place within the following hour. All measurements were performed at a 1.5 T system (Magnetom Avanto, Siemens) in combination with a 4 + 4 channel multifunctional coil array (NORAS MRI products). The relaxation time constant *T*_1_ was obtained through a segmented 2D IRSnapshotFlash method (*T*_R_/*T*_E_ = 8.7 ms/4.8 ms, matrix: 256 × 176, inplane resolution: 0.7 × 0.7 mm^2^, slice thickness: 20 mm, number of segments: 44, number of echoes: 128, *T*_A_ = 4.75 min) [45–46]. Image reconstruction, data fitting and a manually segmentation of the tubes was done offline using Matlab R2012b (The Mathworks). The program used for the statistical analysis was PASW Statistics 18 (IBM).

### Biocompatibility

CaF_2_:(Tb^3+^,Gd^3+^) NPs were stabilized by shaking the particles in a 20 vol % Melpers^®^2450 dispersion in water for 18 h. Afterwards the NPs were centrifuged, washed two times with DI water and finally redispersed in DMEM with 10% FCS.

The sedimentation studies over 24 h were carried out by monitoring the absorbance at 700 nm as a function of time (Shimadzu, UV-3100). The sample (5 mg·mL^−1^ NPs in DMEM with 10% FCS, with or without Melpers^®^2450) was placed into a polystyrene micro cuvette and the absorbance was measured in 20 min time intervals. As the measurement beam entered the cuvette approximately 1.5 cm from the bottom of the cuvette, the supernatant of the sedimenting sample was measured. As a reference DMEM with 10% FCS was used. The first measurement was taken as 1.0 and the reference as 0.

The cell toxicity of the CaF_2_:(Tb^3+^,Gd^3+^) NPs was investigated in 96-well plates on a subconfluent monolayer culture of hdF. With the CellTiter-Glo luminescent cell viability assay (Promega), based on the quantification of the ATP concentration, the cell viability was examined. The cell line was seeded into 96-well cell-culture plates at a number of 1.47·10^4^ cells per square centimeter. Dilutions of CaF_2_:(Tb^3+^,Gd^3+^) NP samples in the concentration range of 0.5–1 mg·mL^−1^ in DMEM with 10% FCS were added in triplicate. Wells containing 10% sodium dodecyl sulfate (SDS) and untreated hdF in DMEM with 10% FCS were used as positive and negative control, respectively. After 24 h of incubation, the CellTiter-Glo reagent was administered per well according to the instructions of the manufacturer. Briefly, the test solutions were removed by washing with PBS buffer, the cells in each well were overlaid with 100 μL of basal medium and 100 μL of CellTiter-Glo reagent and luminescence was measured after two minutes of shaking and ten minutes incubation at room temperature in a TECAN plate reader (infinite M200, TECAN, Maennedorf, Switzerland). According to DIN EN ISO 10993-5, a more than 20% deviation of measurement values of treated cells compared to the untreated control was defined as cytotoxic.

## Supporting Information

File 1 Additional figures and data.
