# Supplementary material for: Calcium fluoride based multifunctional nanoparticles for multimodal imaging
Source: Beilstein J Nanotechnol. 2017 Jul 18;8:1484–93. doi: 10.3762/bjnano.8.148 (PMC5530611; doi:10.3762/bjnano.8.148)
Supplement: File 1 — Additional figures and data. [file Beilstein_J_Nanotechnol-08-1484-s001.pdf]

Supporting Information

for

Calcium fluoride based multifunctional nanoparticles for multimodal imaging

Marion Straßer*,‡,1,2, Joachim H. X. Schrauth‡,3,4, Sofia Dembski[1,5,6], Daniel Haddad[3,7], Bernd Ahrens[8,9], Stefan Schweizer[8,9], Bastian Christ[5], Alevtina Cubukova[5], Marco Metzger[5,6], Heike Walles[5,6], Peter M. Jakob[3,4] and Gerhard Sextl[1,2]

Address: [1]Fraunhofer Institute for Silicate Research ISC, Neunerplatz 2, 97082 Wuerzburg, Germany; [2]Department of Chemical Technology of Materials Synthesis, University of Wuerzburg, Roentgenring 11, 97070 Wuerzburg, Germany; [3]MRB Research Center for Magnetic Resonance Bavaria, Am Hubland, 97074 Wuerzburg, Germany; [4]Department of Experimental Physics 5 (Biophysics), University of Wuerzburg, Am Hubland, 97074 Wuerzburg, Germany; [5]Translational Center Wuerzburg "Regenerative Therapies for Oncology and Musculosceletal Diseases", Branch of Fraunhofer Institute for Interfacial Engineering and Biotechnology IGB, 97070 Wuerzburg, Germany; [6]University Hospital Wuerzburg, Chair Tissue Engineering and Regenerative Medicine, Roentgenring 11, 97070 Wuerzburg, Germany; [7]Magnetic Resonance and X-ray Imaging Department of Fraunhofer Development Center X-ray Technology EZRT, a division of Fraunhofer Institute for Integrated Circuits IIS, Am Hubland, 97074 Wuerzburg, Germany; [8]South Westphalia University of Applied Sciences, Luebecker Ring 2, 59494 Soest, Germany and [9]Fraunhofer Application Center for Inorganic Phosphors, Branch Lab of Fraunhofer Institute for Microstructure of Materials and Systems IMWS, Luebecker Ring 2, 59494 Soest, Germany

Email: Marion Straßer* - marion.strasser@isc.fraunhofer.de

* Corresponding author

‡ Equal contributors

Additional figures and data

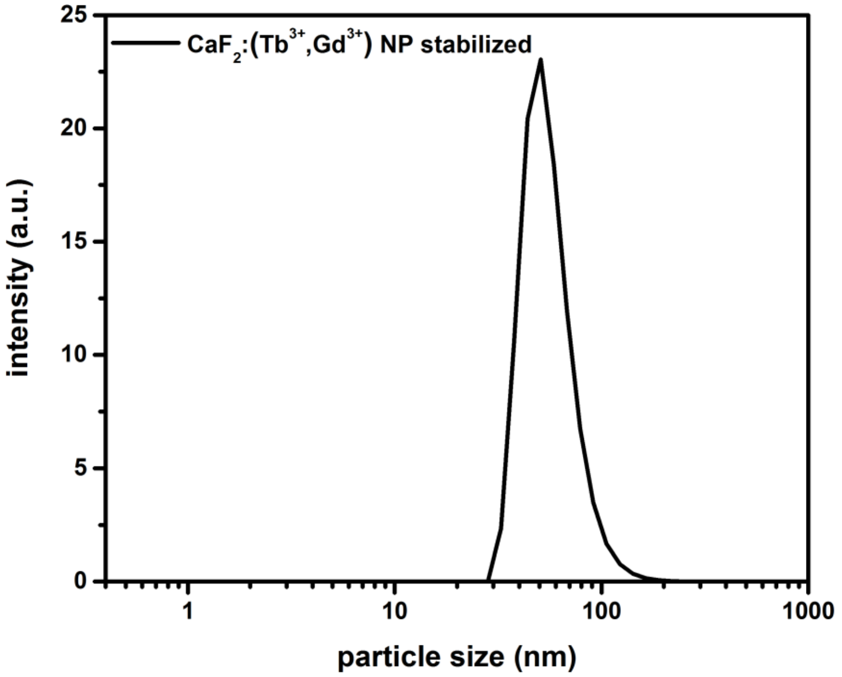

Figure S1: DLS measurement of the CaF_2:(Tb^{3+},Gd^{3+}) NPs stabilized with Melpers®2450 (number-weighted).

$$r_1 = \frac{4.59 \cdot 10^3 \cdot \mathrm{mL} \cdot \mathrm{mol}}{10^{-3} \cdot \mathrm{mol} \cdot \mathrm{s} \cdot 938 \cdot 10^3 \cdot \mathrm{mg}}$$

Relaxivity Magnevist: 4.59 $L \cdot mmol^{-1} \cdot s^{-1}$ from literature [38]

Molar Mass Magnevist: 938 $g \cdot mol^{-1}$

Figure S2: Calculating method for converting the unit of the relaxivity.

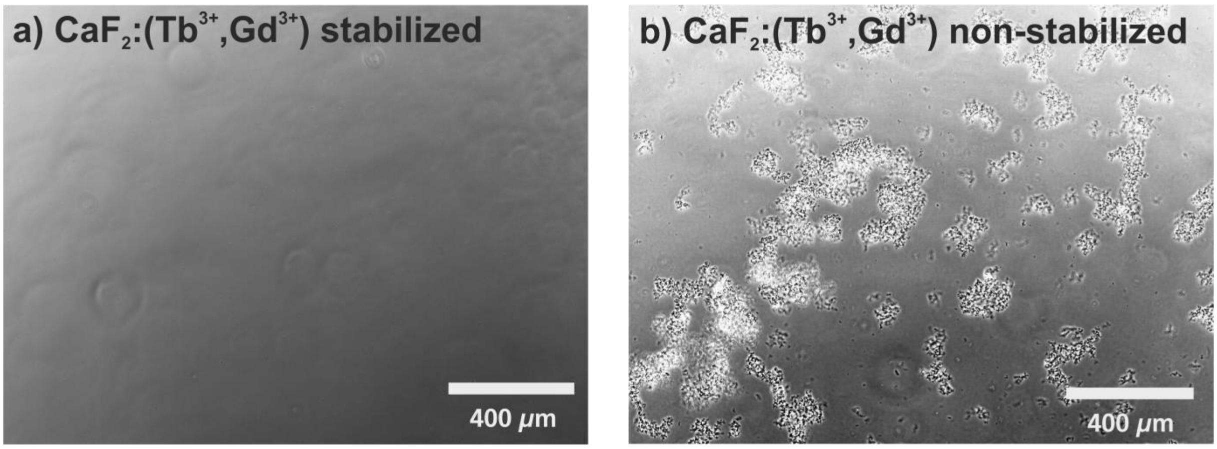

Figure S3: Microscopic images of the CaF_2:(Tb^{3+},Gd^{3+}) NPs (c = 5 mg·mL^{-1}): a) stabilized with Melpers®2450 and b) non-stabilized.

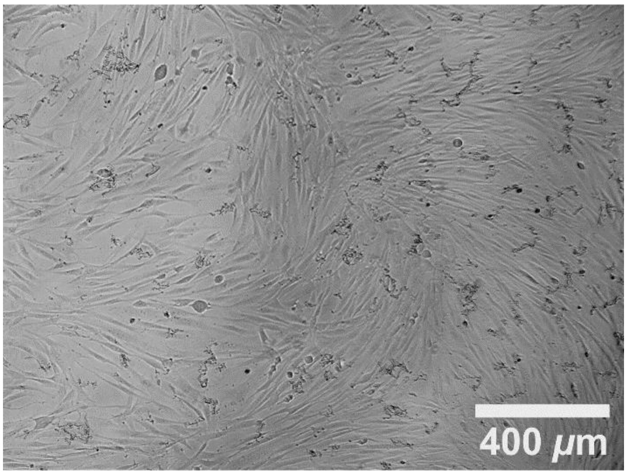

Figure S4: Representative microscopic image of the untreated hdF in DMEM with 10% FCS.
